# Supplementary material for: Microbial Assessment in A Rare Norwegian Book Collection: A One Health Approach to Cultural Heritage
Source: Microorganisms. 2024 Jun 17;12(6):1215. doi: 10.3390/microorganisms12061215 (PMC11206040; doi:10.3390/microorganisms12061215)
Supplement: Supplementary file 1 [file microorganisms-12-01215-s001.zip › microorganisms-3053770-supplementary.pdf]

Microbial Assessment in A Rare Norwegian Book Collection: A One Health Approach to Cultural Heritage

Sílvia O. Sequeira [1,2,*], Ekaterina Pasnak [1], Carla Viegas [3,4,*], Bianca Gomes [3,5], Marta Dias [3,4], Renata Cervantes [3,4], Pedro Pena [3,4], Magdalena Twarużek [6], Robert Kosicki [6], Susana Viegas [4], Liliana Aranha Caetano [3,7], Maria João Penetra [8], Inês Silva [8,9], Ana Teresa Caldeira [8,9] and Catarina Pinheiro [2,8,9,]

Supplementary material

Table S1 - Mass spectrometry parameters for MRM transitions in the negative and positive ion mode monitoring.

		Precursor ion [m/z]	Product ions [m/z] [a]	Declustering potential [V]	Collision energy [V]	Cell exit potential [V]
15-Acetyldeoxynivalenol	[M+H]+	339.1	321.2/137.2	91	13/17	18/8
3-Acetyldeoxynivalenol	[M+Ac]-	397.3	59.2/307.1	-70	-38/-20	-8/-7
Aflatoxin B1	[M+H]+	313.1	285.2/128.1	106	33/91	16/10
Aflatoxin B2	[M+H]+	315.1	287.2/259.2	96	37/43	18/18
Aflatoxin G1	[M+H]+	329.1	243.1/200.0	86	39/59	14/12
Aflatoxin G2	[M+H]+	331.1	313.2/245.2	111	35/43	18/14
Aflatoxin M1	[M+H]+	329.1	273.2/229.1	91	35/59	16/12
a-Zearalanol	[M-H]-	321.2	277.2/303.2	-115	-32/-30	-13/-15
a-Zearalenol	[M-H]-	319.2	160.1/130.1	-115	-44/-50	-13/-20
b-Zearalanol	[M-H]-	321.2	277.2/303.2	-115	-32/-30	-13/-15
b-Zearalenol	[M-H]-	319.2	160.0/130.0	-115	-44/-50	-13/-20
Deepoxydeoxynivalenol	[M+Ac]-	339.1	59.1/249.0	-70	-20/-18	-9/-17
Deoxynivalenol	[M+Ac]-	355.1	265.2/59.2	-70	-22/-40	-13/-8
Diacetoxyscirpenol	[M+NH4]+	384.2	307.2/105.1	81	17/61	9/7
DON Glucosid	[M+Ac]-	517.3	427.1/59.1	-80	-30/-85	-11/-7
Fumonisin B1	[M+H]+	722.5	334.4/352.3	121	57/55	4/12
Fumonisin B2	[M+H]+	706.5	336.4/318.4	126	59/51	8/2
Fumonisin B3	[M+H]+	706.5	336.3/318.5	126	59/51	8/2
Fusarenon-X	[M+Ac]-	413.2	59.1/263.0	-70	-44/-22	-9/-16
Gliotoxin	[M+H]+	327.1	263.2/245.3	61	15/25	16/20
Griseofulvin	[M+H]+	353.2	165.2/215.2	81	27/27	10/12
HT-2 Toxin	[M+NH4]+	442.2	263.1/345.1	76	21/27	19/20
Mevinolin	[M+H]+	405.3	199.2/173.3	76	17/29	14/10
Moniliformin	[M-H]-	96.9	41,2	-100	-24	-5
Monoacetoxyscirpenol	[M+NH4]+	342.2	265.1/307.2	71	13/13	26/8
Mycophenolic acid	[M+NH4]+	338.1	207.2/303.2	61	33/19	16/18
Neosolaniol	[M+NH4]+	400.2	215.0/185.0	76	25/29	12/14
Nivalenol	[M+Ac]-	371.1	281.1/59.1	-75	-22/-45	-15/-7
Ochratoxin A	[M+H]+	404.0	239.0/102.0	91	37/105	16/14
Ochratoxin B	[M+H]+	370.1	205.0/103.1	86	33/77	12/16
Patulin	[M-H]-	153.0	109.0/81.0	-50	-12/-18	-9/-11
Roquefortine C	[M+H]+	390.2	193.2/322.2	91	39/29	10/18
Sterigmatocystin	[M+H]+	325.1	310.2/281.1	96	35/51	18/16

T2-Tetraol	[M+NH4]+	316.2	215.2/281.2	61	13/13	16/8
T2-Toxin	[M+NH4]+	484.3	215.2/185.1	56	29/31	18/11
T2-Triol	[M+NH4]+	400.2	281.3/215.2	71	13/17	16/12
Zearalanone	[M-H]-	319.2	205.2/107.0	-125	-34/-40	-13/-5
Zearalenon	[M-H]-	317.1	131.1/175.0	-110	-42/-34	-8/-13

[a] the ions are presented in quantitative and qualitative order
